# The use of COPD maintenance therapy following spirometry in General Practice

**DOI:** 10.3402/ecrj.v3.30232

**Published:** 2016-06-22

**Authors:** Vibeke Gottlieb, Anne Marie Lyngsø, Ditte Sæbye, Anne Frølich, Vibeke Backer

**Affiliations:** 1 Respiratory Research Unit, Department of Respiratory Medicine, Bispebjerg University Hospital, Copenhagen, Denmark; 2 Research Unit for Chronic Conditions, Bispebjerg and Frederiksberg University Hospital, Copenhagen, Denmark; 3 Institute of Preventive Medicine, Frederiksberg University Hospital, Frederiksberg, Denmark

**Keywords:** COPD, screening, treatment

## Abstract

**Background:**

Several studies have shown that the use of pulmonary medication is widespread and often initiated without initial spirometry. Early detection of chronic obstructive pulmonary disease (COPD) by spirometry in General Practice is essential for an early and correct implementation of medical treatment.

**Aim:**

The aim of the present study was to evaluate the use of regular therapy following diagnostic spirometry for COPD in General Practice from February 2008 to February 2009.

**Method:**

Spirometry data and results were linked through Statistics Denmark with information from the Register of Medicinal Product Statistics using the unique personal identification code. Data were analysed to evaluate the impact of screening on use of regular COPD therapy. Primary outcome was initiation of regular therapy following COPD diagnosis with spirometry.

**Results:**

In a population of 3,376 individuals at risk, 1,458 underwent spirometric assessment with 631 being diagnosed with COPD; 110 of those received regular therapy before assessment with this figure increasing to 161 after spirometry. Of 827 participants not receiving a COPD diagnosis, 36 received regular therapy prior to assessment and 42 received regular therapy after spirometry despite no established COPD diagnosis.

**Conclusion:**

There is a significant chance of receiving regular therapy after being diagnosed with COPD. However, a large proportion of subjects diagnosed with COPD did not receive regular therapy following diagnosis. Efforts should be made to ensure correct diagnosis and correct medical treatment according to guidelines in individuals with COPD.

It is estimated that by the year 2020 chronic obstructive pulmonary disease (COPD) will be among the leading causes of death worldwide. COPD is an increasing burden on society (1, 2), constituting one of the primary causes of morbidity and mortality worldwide.

Early detection of COPD by diagnostic spirometry is essential in reducing the impact of disease. There may be a rationale behind the treatment of individuals with mild and moderate COPD, as exercise impairment tends to be evident even in mild disease (3–5), in part due to early physiological changes leading to dynamic hyperinflation.

Regarding the effect of pharmacotherapy in mild COPD, the GOLD guidelines (6) acknowledge that there has been little evidence to support the prescription of bronchodilation therapy in mild disease. However, several studies show the existence of activity-related dyspnoea in mild disease (7, 8), and other studies are now examining the effects of bronchodilation in specific relation to the physiological changes of expiratory airflow limitation leading to gas trapping and reduced inspiratory capacity (9).

Pharmacological treatment of COPD has been associated with sustained improvement in symptoms, exercise capacity, overall health status, and a reduction in frequency of exacerbations and possibly in the rate of decline of lung function (10, 11). In mild disease (GOLD stage 1), there is evidence of a physiological effect of medical treatment with reduced lung hyperinflation at rest and during treadmill exercise, and in individuals with moderate airflow obstruction (GOLD stage 2) with the further effect of an increase in exercise tolerance (12).

The majority of individuals with COPD are handled in general practice and studies have shown that adherence to treatment guidelines vary (13, 14).

The standard therapy for persons with COPD has been described in a number of guidelines (15–17), of which the GOLD strategy (6) remains the most prevalent. Inhalation therapy is often prescribed only on the basis of symptoms, the result being that a large number of individuals with undetected COPD do, when diagnosed, receive some degree of therapy.

The aim of the present study is to evaluate the prescription of guideline-based regular inhalation therapy following a COPD screening project in General Practice (18). The focus of the present study has been individuals at risk of COPD prior to the screening process and on those diagnosed with COPD during the screening process.

## Materials and methods

### Study design

The study was designed as a registry-based study using Danish National Registry Databases. The registry used for the study was the Danish Register of Medicinal Product Statistics (here termed prescription register NPR) which contains all information on dispensed prescriptions from Danish pharmacies since 1995 where drugs are classified according to the Anatomical Therapeutic Chemical (ATC) system. As the government-financed healthcare system partially reimburses drug expenses, all Danish pharmacies are obliged to register dispensed drug prescriptions ensuring a valid and accurate register (19).

### Population

**Fig. 1:**
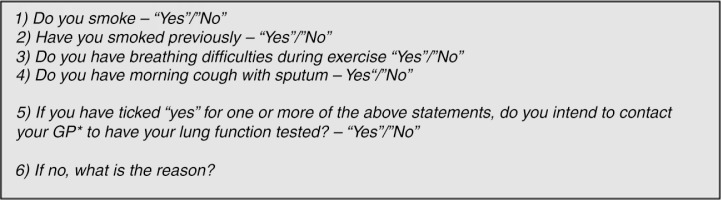
Questionnaire used for the screening model.

The data used in this study originated from the Copenhagen COPD screening project (18). This project was designed to test a screening model for COPD in General Practice. The study was performed from February 2008 to February 2009 among individuals aged 65+ years in a central area of Copenhagen. A total of 7,103 persons received written information and an invitation – with a subsequent response rate of 81%. Figure 1 illustrates the questionnaire used for risk assessment.

Participants were asked to indicate whether they were smokers, former smokers, or had daily symptoms in the form of dyspnoea or cough with phlegm. On the basis of the responses, a risk assessment of each respondent was made with 3,376 being at risk of COPD. Individuals at risk of COPD were asked to attend their general practitioner (GP) for a diagnostic spirometry. The persons considered in the present survey were those at risk of COPD, based on responses to the initial questionnaire as shown in Table 1. That is, smokers, former smokers, or subjects experiencing daily symptoms. Respondents at risk were asked to contact their GP for spirometric assessment. Spirometries were carried out by 35 GPs of that specific area of Copenhagen, and by the study nurses at the healthcare centres of 10 GPs not being able to do spirometric assessments themselves. Spirometric results were handled by the GP of the participants.

**Table 1:** Risk assessment for respondents (18)

	Symptoms *N* (%)	No symptoms *N* (%)	Unknown *N* (%)	Total *N* (%)
Current smoker	461 (49.1)	356 (38.0)	121 (129)	**938 (16.31)**
Former smoker	838 (41.5)	1,016 (50.3)	166 (8.2)	**2,020 (35.0)**
Never smoker	**374 (17.2)**	1,761 (81.2)	35 (1.6)	2,170 (37.6)
Smoking status unknown	**44 (6.9)**	8 (1.3)	587 (91.9)	639 (11.1)
Total	1,717 (29.8)	3,141 (54.5)	909 (15.7)	5,767 (100)

Subjects at risk are given in bold numbers.

### Study protocol

The database study included all individuals from the initial screening project (18). These were linked with data from the Danish Register of Medicinal Product Statistics using the unique Danish personal identification number (19).

Data on ATC codes R03 was used for all included.

The use of regular therapy was assessed at baseline, defined as a 2-year period prior to the first screening event.

The use of medication prior to the diagnostic spirometry was taken into account when evaluating the impact of spirometric assessment.

1. Individuals at risk of COPD and receiving regular therapy prior to spirometry.
2. Individuals at risk of COPD and receiving no regular therapy prior to spirometry.

### Definition

As the use of inhalation therapy was limited, individuals were considered as receiving any inhalation therapy or no inhalation therapy.

Relevant regular therapy comprised long-acting beta-agonists (LABA), long-acting anticholinergics, LAMA, and fixed combination of ICS and LABA/LAMA therapy.

Rescue medication comprised short-acting beta agonist (SABA) and was only assessed at baseline.

### Statistics

Data management and data analysis were performed using the SAS statistical package version 9.3.

Baseline was assessed as mean±SD.

The non-parametric Kruskal–Wallis test was performed for all of the continuous outcome variables between the severity groups of COPD.

Chi-square test was performed for the test of independence between categorical variables and the GOLD classification.

Regular therapy for each individual was assessed over a 2-year period prior to spirometry and for 2 years following spirometric assessment.

The use of as needed medication was assessed as ‘none’ or ‘any’ prior to spirometric assessment.

GOLD stage 1–4 was defined according to the level of lung function and the GOLD strategy from 2011 (13).

## Results

A total of 1,458 individuals underwent spirometric assessment, with 631 receiving a COPD diagnosis. Data concerning these subjects have been linked with the Danish National Prescription Registry.

The 631 participants with COPD were primarily diagnosed with mild to moderate disease.

Table 2 illustrates the distribution of subjects according to spirometric results and baseline characteristics. Non-parametric Kruskal–Wallis test shows a highly significant difference between the groups for all of the continuous outcome variables (*p*<0.0005). Chi-square test for independence between categorical variables and GOLD classification led to highly significant dependence with severe COPD receiving maintenance and rescue therapy.

**Table 2:** Baseline characteristics

		GOLD classification			
		
	No COPD	GOLD 1	GOLD 2	GOLD 3	GOLD 4
Number (*n*)	827	269	271	84	7
Age (SD)	73.6 (6.2)	74.6 (6.8)	75.2 (7.1)	75.9 (6.8)	77.3 (5.0)
Male (*n*)	371	80	129	37	3
FEV1 (SD)	2.34 (0.68)	2.17 (0.54)	1.66 (0.48)	1.01 (0.28)	0.65 (0.17)
FEV1% (SD)	96.1 (19.3)	97.3 (12.7)	67.2 (9.2)	41.7 (5.5)	27.7 (3.3)
FVC (SD)	3.03 (0.89)	3.41 (0.89)	2.82 (0.78)	2.02 (0.60)	1.63 (0.50)
FVC% (SD)	98.3 (21.3)	121.5 (19.6)	89.2 (15.7)	66.0 (14.1)	56.1 (13.5)
FEV1/FVC (SD)	77.6 (5.6)	64.1 (4.7)	59.3 (7.3)	51.4 (9.7)	41.1 (8.1)
Regular therapy (*n*)	36	17	50	39	4
As needed (*n*)	43	16	33	32	4

Use of regular therapy was assessed for individuals with COPD and for those not being diagnosed with COPD following spirometric assessment.

Table 3 illustrates that one fifth of the participants diagnosed with COPD receive some level of maintenance therapy before spirometric assessment and this figure increases to one-fourth after spirometry.

**Table 3:** Prescription of regular therapy

	Before	After
COPD (chi-square test *p*<0.001)		
No therapy	521	470
Therapy	110	161
**Total**	**631**	**631**
No COPD (chi-square test *p=*0.6)		
No therapy	791	785
Therapy	36	42
**Total**	**827**	**827**

A small number of participants not diagnosed with COPD do in fact receive pulmonary medication. Of 827 persons undergoing spirometric assessment and not receiving a COPD diagnosis, 36 continue to receive regular therapy and six subjects not receiving a COPD diagnosis are subsequently prescribed regular therapy.

## Discussion

In the present survey, we demonstrated that among a large group of patients at risk of COPD, 10% received inhalation therapy prior to spirometric assessment. Following spirometric assessment and COPD diagnosis, there was only a slight increase in the use of regular therapy.

The purpose of early detection of COPD is to initiate treatment, pharmacological and non-pharmacological, as early as possible, bearing in mind that the burden of morbidity associated with COPD is substantial, even prior to diagnosis (20–22). An early intervention is thus aimed at reducing this burden – limiting the future costs (23) and at promoting quality of life for the individuals affected.

Initiating medical treatment can be important and is recommended, but equal in importance is advice on smoking cessation, an active lifestyle and in some cases pulmonary rehabilitation (6).

One year prior to initiation of the present study a total of 124 spirometries were performed among individuals aged 65+ years in the same area of Copenhagen (unpublished data), highlighting the need for action.

Several studies have previously emphasised the limited use of spirometry in General Practice. Matheson et al. showed that of 1,224 subjects undergoing spirometric assessment, 40% of subjects with COPD did not have a diagnosis from their GP and only 48.7% of those with COPD had ever been prescribed medication for their breathing (24–26). A recent study among GPs points to the fact that when not performing spirometry, physicians are likely to underestimate disease severity. In this particular study, the use of spirometry resulted in change of treatment for approximately one-third of the patients (27). The limited use of spirometry does not necessarily correlate with limited use of pulmonary therapy. Treatment with inhalation therapy is often initiated without spirometric assessment. This finding is similar to the findings by Lange et al., showing in the Danish KVASIMODO study that a diagnosis of COPD and subsequent treatment is supported by spirometric data in only 50% of the cases (14).

The majority of individuals diagnosed during the screening process, a total of 540, had mild to moderate COPD, and it is therefore plausible to assume that this group had not previously consulted their GPs with symptoms of dyspnoea or cough, as studies have shown that action is only taken when symptoms become a severe everyday limitation (28, 29). However, despite this assumption, only minor change is seen in the number of subjects receiving therapy. Prior to spirometry, a total of 110 subjects received regular therapy; after spirometric assessment this number of subjects increased by 51 to a total of 161 subjects with COPD being prescribed regular therapy. This means that a large number of subjects with mild to moderate COPD did not receive regular therapy and are therefore, to a large extent, not treated in accordance with guidelines contributing to similar previous findings (25).

Initiating any kind of medical treatment always requires a combination of adherence to guidelines and considerations regarding the individual patient (30–32). In the current study, there was a substantial focus on COPD. Individuals undergoing spirometry voluntarily contacted their GP and thus expressed an interest in taking care of this aspect of their own health. As the spirometries were part of a screening process the GPs and their staff took part in lessons on spirometry as well as on current treatment guidelines (1, 18, 33). In light of this, it makes little sense that the use of pulmonary medication is not made use of following a diagnosis.

Initiating treatment should of course be carefully considered in any setting – and there are many considerations to take into account – but knowing that individuals with mild to moderate COPD can experience limitations in their everyday life and that there are distinct physiological changes present at early stages of disease, the initiation of treatment for this group of patients should indeed be considered (7, 8, 22).

Treatment recommendations for COPD today include SABAs for all individuals diagnosed with COPD, even those without symptoms. The number of individuals receiving treatment according to guidelines may therefore be higher than what we can account for as we only have baseline information on as needed therapy.

The study involved 45 general practices, in central Copenhagen – one reason for the discrepancy between spirometric results and treatment may therefore be that the changes concerning maintenance medication actually seen are in fact due to the action of a few GPs treating their patients according to guidelines. The average GP performed 25, ranging from 0 to 51, spirometries throughout the study. The project nurse performed around 500 spirometries for the GPs not able to perform spirometric assessments themselves (unpublished data).

No record was made of prior participation in smoking cessation programmes or in pulmonary rehabilitation.

The present study is only part of a larger screening study focusing on developing a COPD screening programme in the primary care sector. Individuals with moderate COPD were referred to pulmonary rehabilitation in the municipality. For individuals diagnosed with mild disease – GOLD stage 1 – advice on physical activity and smoking cessation was on the discretion of the GP – and no specific follow-up was done.

A follow-up of the population regarding degree of smoking cessation and daily activity would indeed be interesting; however, this was not within the scope of our investigation.

It has been reported that the attendance rate to pulmonary rehabilitation is generally low as individuals with COPD are often not referred and as rehabilitation programmes offered do not cover the required needs of the individuals who are actually referred (34), despite the fact that pulmonary rehabilitation has been shown to have a significant effect on physical exercise capacity and health-related quality of life (35, 36).

## Conclusion

Although some effect is seen, it is essential that the use of spirometry as a diagnostic tool be followed by relevant treatment of individuals diagnosed with COPD. Other studies have similarly shown that treatment in accordance with guidelines vary at best (25–27). Even when making the correct diagnosis of COPD in a patient, this diagnosis in itself is of no importance when it is not followed by correct treatment in terms of advice on smoking cessation, advice on physical activity, medical treatment and, in some cases, pulmonary rehabilitation.

In conclusion, our survey highlights the problems associated with early detection of COPD in general practice. It was possible, in the setting of a screening project, to perform spirometries in almost half of the individuals at risk of COPD. However, only in 10% of individuals diagnosed with COPD maintenance medical treatment was initiated. Considering that 362 participants were identified as having moderate to very severe COPD, the result is surprising as evidence on the importance of early treatment is increasing. The use of inhalation therapy provides symptomatic relief and increases the likelihood of a high level of daily physical activity.
